# Raman fingerprint for semi-metal WTe_2_ evolving from bulk to monolayer

**DOI:** 10.1038/srep19624

**Published:** 2016-01-22

**Authors:** Y. C. Jiang, J. Gao, L. Wang

**Affiliations:** 1 Research Center for Solid State Physics and Materials, School of Mathematics and Physics, Suzhou University of Science and Technology, Suzhou 215009, China; 2 Department of Physics, The University of Hong Kong, Pokfulam Road, Hong Kong; 3 School of Materials Science and Engineering, Shanghai University, 333 Nanchen Road, Baoshan District, Shanghai 200444, China

## Abstract

Tungsten ditelluride (WTe_2_), a layered transition-metal dichalcogenide (TMD), has recently demonstrated an extremely large magnetoresistance effect, which is unique among TMDs. This fascinating feature seems to be correlated with its special electronic structure. Here, we report the observation of 6 Raman peaks corresponding to the 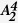, 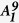, 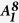, 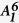, 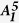 and 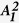 phonons, from the 33 Raman-active modes predicted for WTe_2_. This provides direct evidence to distinguish the space group of WTe_2_ from those of other TMDs. Moreover, the Raman evolution of WTe_2_ from bulk to monolayer is clearly revealed. It is interesting to find that the 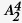 mode, centered at ~109.8 cm^**−**1^, is forbidden in a monolayer, which may be attributable to the transition of the point group from *C*_*2v*_ (bulk) to *C*_*2h*_ (monolayer). Our work characterizes all observed Raman peaks in the bulk and few-layer samples and provides a route to study the physical properties of two-dimensional WTe_2_.

Transition-metal dichalcogenides (TMDs) of the MX_2_ type, where M refers to Mo and W, and X refers to S, Se and Te, have attracted much attention as graphene-like semiconductors with remarkable electric, spintronic and optical properties^1^,^2^,^3^,^4^,^5^,^6^,^7^. The basic structure of bulk MX_2_ comprises two-dimensional (2D) X-M-X layers stacked through Van der Waals forces. Mono- and few-layer MX_2_ can be fabricated by the mechanical exfoliation method^8^. The physical properties of MX_2_ are greatly dependent on the number of layers^9^,^10^,^11^. Weak Van der Waals forces play a very important role in the interlayer bands and atomic vibration. As the thickness decreases, MX_2_ exhibits a transition from indirect to direct gaps, while the frequency of the Raman peaks shift away from those of the bulk sample^12^,^13^.

Tungsten ditelluride (WTe_2_) is a unique TMD as it has the large interlayer spacing, an orthorhombic lattice structure and a semi-metal electronic structure^1^,^14^,^15^,^16^. This material has drawn extensive interest since the recent discovery of an extremely large magnetoresistance (XMR) effect in diamagnetic WTe_2_ single crystal, which has not been observed in other TMDs^1^. Such a fascinating feature implies potential spintronic applications for mono- and few-layer WTe_2_. However, to date no study has examined the physical properties of the 2D WTe_2_ system. Raman spectroscopy is a powerful tool for investigating the symmetries of 2D semiconductors; it is necessary, therefore, to characterize the Raman fingerprint of WTe_2_ as it evolves from a 3D to a 2D system.

This article reports the observation of Raman scattering in bulk and few-layer WTe_2_ samples. Six optical vibrational modes, 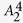, 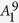, 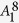, 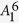, 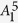 and 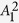, were observed at room temperature. Density functional perturbation theory (DFPT) was used to calculate phonon branches, simulate Raman spectra and verify their corresponding optical phonons. Group analysis demonstrated that the lattice symmetry differentiated WTe_2_ from other TMDs^17^,^18^. In our experiments, WTe_2_ flakes, exfoliated from a piece of bulk, showed strong thickness-dependent Raman spectra. As the number of layers increased, the 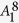, and 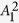 modes softened (red-shift) and the 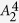 and 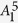 mode stiffened (blue-shift). It was also interesting to find that the 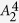 mode was absent in the monolayer. This phenomenon may be attributable to the transition of the point group from *C*_*2v*_ to *C*_*2h*_. Our results provide a well-defined and reliable method for determining the number of layers using Raman spectroscopy.

## Results and Discussions

**Figure 1:**
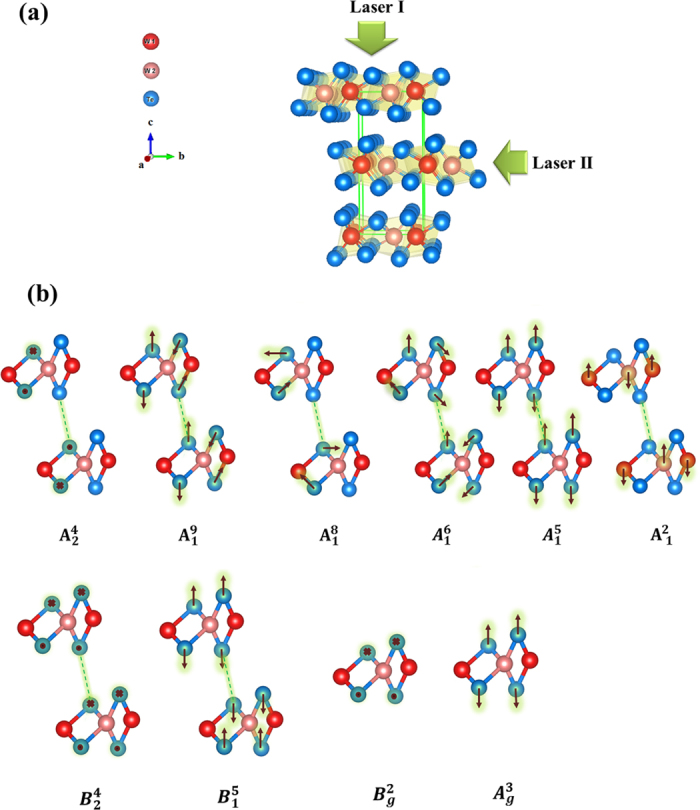
Lattice structure. (**a**) Crystal structure of WTe_2_ along the a-axis direction, showing the orthorhombic unit cell. (**b**) Atomic displacements of Raman active modes in WTe_2_ bulk and monolayer. Here, “×” and “·” indicate that the Te atoms move into and out of the *bc* plane, respectively.

Figure 1a shows the layered crystal structure of WTe_2_, in which tungsten layers are sandwiched between two neighboring tellurium sheets. The distance (0.703 nm) between two adjacent sandwich layers is the largest of all known TMDs. Most TMDs exhibit a hexagonal structure with the space group *D*^*4*^_*6h*_. However, naturally formed WTe_2_ reveals an orthorhombic unit cell with the space group *C*^*7*^_*2v*_ (*Pmn*2_1_), which may be derived from a distorted hexagonal net^1^,^15^. The unit cell contains two tungsten atoms and four tellurium atoms. The two tungsten atoms (distinguished by W1 and W2) are not equivalent to each other and the W-Te bond length ranges from 2.7 to 2.8 Å. WTe_2_ bulk exhibits the T_d_ stacking order, in which the atoms in the upper layer are rotated by 180 degrees with respect to those in the lower layer^17^. And the tungsten atoms shift away from the center of the octahedron formed by the tellurium atoms. Therefore, the vibrational modes of Td-WTe_2_ are very different from those of 2H-MX_2_, and the previous calculation of vibrational modes, based on 2H-WTe_2_, is inapplicable to our experimental Raman results^19^.

Due to the *C*^*7*^_*2v*_ symmetry group, the irreducible representations of the optical phonons in the bulk WTe_2_ at the center of the Brillouin zone (*Γ* point) are


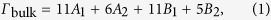


where all of the vibrational modes are Raman active, and the 11A_1_, 11B_1_ and 5B_2_ modes are infrared active. Here, the Raman tensors corresponding to different symmetries can be written as


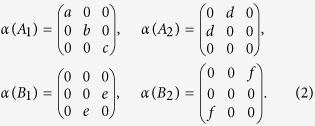


Although 33 Raman vibrations are predicted by group theory, only 6 Raman peaks were observed in our experiments. To distinguish these vibrational modes, we use 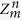 (Z = A or B; m = 1 or 2; *n* = integer) to represent them. The frequency of 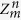 mode is smaller than that of 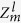 mode if *n* is larger than *l*. This method allows us to name all appropriate vibrational modes.

**Figure 2:**
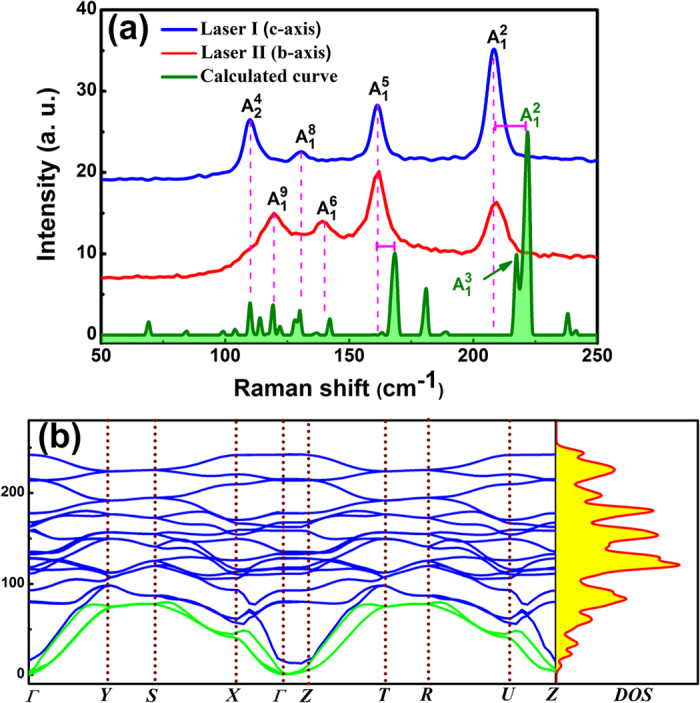
Experimental and calculated phonons. (**a**) Raman spectra of the bulk sample with incident Laser I and Laser II along the *c*- and *b*-axes at room temperature, respectively. The calculated Raman spectrum is plotted for comparison. (**b**) Calculated phonon dispersion curve along the *Γ-Y-X-Γ-Z* direction in the orthorhombic Brillouin zone (BZ), and the vibrational density of states (DOS) for WTe_2_ bulk at the equilibrium volume. The blue and green curves are the optical and acoustic vibrational branches, respectively.

DFPT has been used to calculate all 33 Raman vibrational modes. Among them, the atomic displacements of the six Raman active modes that could potentially explain our Raman results are shown in Fig. 1b. The Raman spectra of WTe_2_ bulk at room temperature are presented in Fig. 2a. Raman peaks centered at ~109.8 cm^**−**1^, ~130.4 cm^**−**1^ ~162.5 cm^**−**1^ and 208.4 cm^**−**1^ were observed with Laser I applied in the *z(xx)*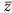 geometry. Another two Raman peaks, centered at ~119.6 cm^**−**1^ and ~139.1 cm^**−**1^, were observed with Laser II applied in the *y(zz)*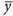 geometry. Due to the non-polarized Raman measurement, the 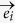 and 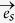 vectors are not along a- or c- axis in the either geometry. Thus, a total of six Raman peaks were identified in our experiments. Due to the non-polarized Raman measurements, the assignment of these Raman peaks could not be performed through symmetry analysis. Instead, we calculated the Raman intensities and spectrum (green curve in Fig. 2a) to verify the phonon modes corresponding to the observed Raman peaks. Only 6 Raman peaks were observed in comparison with 33 Raman modes in theory, which suggests that only those modes with high Raman intensities might appear experimentally. For example, Table 1 shows that for the first Raman peak (centered at ~109.8 cm^**−**1^), the 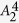, 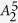 and 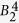 modes were all candidates, with frequencies close to ~110 cm^**−**1^. Only the 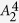 mode exhibited a sufficiently strong Raman intensity, whereas the intensities of the others were very weak. Therefore, the 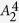 mode should be assigned to the first peak. Similarly, the 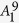 (~119.6 cm^**−**1^),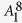 (~130.4 cm^**−**1^), 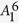 (~139.1 cm^**−**1^), 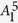 (~162.5 cm^**−**1^) and 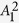 (~208.4 cm^**−**1^) modes were assigned frequencies from low to high, which agrees with the results in ref. 20. It should be noted that although the 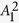 mode was associated with the peak centered at ~208.4 cm^**−**1^, the 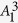 mode still exhibited a strong Raman intensity theoretically and could not be neglected. It is very possible that the 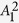 and 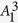 modes both exist, but the difference between their frequencies is so small that they cannot be distinguished in the Raman spectra.

As shown in Fig. 1b, the first five modes originate from the relative movements of Te atoms, while the 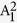 mode is due to the displacement between adjacent W1 and W2 atoms. 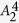 and 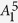 are pure longitudinal and transverse optical modes, respectively. For the in-plane configuration [*z(xx)*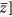, the B_1_ and B_2_ modes are forbidden due to the forms of their Raman tensors. In the case of the out-of-plane configuration [*y(zz)*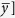, the 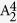 mode may be forbidden and the 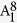 Raman intensity vanishes. Instead, the 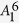 and 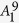 modes reveal significant Raman intensities. Here when we state that one mode is “forbidden”, it means that this Raman mode is prevented from observing by group theory or unable to exist on account of the DFPT calculation, while “vanish” means a Raman mode is active in theory, but in a certain configuration its intensity is too weak to observe due to the specific parameters in its Raman tensor. To clarify this phenomenon, the relationship between Raman intensity and Raman tensor can be written as^21^


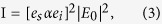


where *e*_*s*_ and *e*_*i*_ are the scattered light and incident light vectors, respectively. This equation makes it easy to understand the anisotropy of the 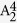 mode. The specific values of a, b and c in the Raman tensors of the 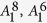 and 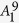 modes determine the dependence on the laser configuration. In the case of Laser I, the 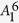 and 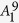 modes with very small values of a and b vanish, while the 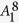 mode appears due to its large value of b. There is a similar effect for Laser II: the 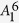 and 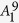 modes appear due to their large c values and the 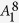 mode vanishes due to its very small a and c values. The a, b and c values of the 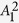 and 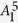 modes are large enough for them to be observed in both configurations.

Figure 2b shows the calculated phonon dispersion curve in the Brillouin zone and the density of states for the WTe_2_ bulk. There are 3 acoustic (green curves) and 33 optical (blue curves) phonon branches. Table 1 shows all of the calculated and experimental vibrational modes. The calculated Raman intensities of the 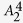, 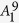, 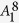, 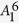, 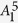 and 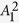 modes are strong at frequencies of ~110.0 cm^**−**1^, ~119.4 cm^**−**1^, ~130.0 cm^**−**1^, ~142.3 cm^**−**1^, ~168.5 cm^**−**1^ and 222.2 cm^**−**1^, respectively. The good agreement between the experimental and theoretical results supports our assignment of the Raman vibrational modes. It should be noted that all calculations were performed at zero temperature, while the Raman spectra were investigated at room temperature. Although frequencies of phonon modes depend on the temperature, it is unable to influence the assignment of the Raman modes. The frequency displacements between 5 K and 294 K were less than 4 cm^**−**1^ for all of the observed Raman peaks^20^. Such small displacements would not affect the association with specific phonon modes. In addition, the frequencies of all Raman peaks increase as the temperature increases, which may explain why most of the calculated frequencies were higher than those measured in the experiments.

**Figure 3:**
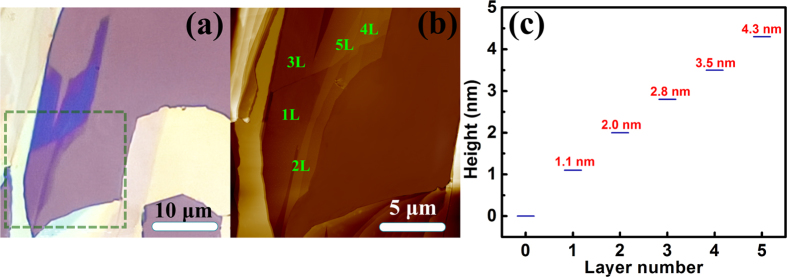
Microscopic Imaging. (**a**) Optical microscopic images of mono- to multilayer WTe_2_ on a Si substrate with a 300 nm SiO_2_ layer. (**b**) AFM image of the area (20 × 20 μm^2^) surrounded by a green dashed line in (**a**). (**c**) Height of the flake as a function of the layer number.

WTe_2_ flakes were fabricated on a Si substrate with a 300 nm SiO_2_ layer using the mechanical exfoliation method. As shown in Fig. 3a,b, an ultrathin WTe_2_ flakes was first identified by optical microscopy, and then imaged by atomic force microscopy (AFM) to determine its thickness. Figure 3c shows that the height of a monolayer WTe_2_ on the SiO_2_ layer is about 1.1 nm, while the height of the monolayer on the WTe_2_ flake is 0.7~0.9 nm and close to the interlayer spacing (about 0.703 nm) of the bulk.

**Figure 4:**
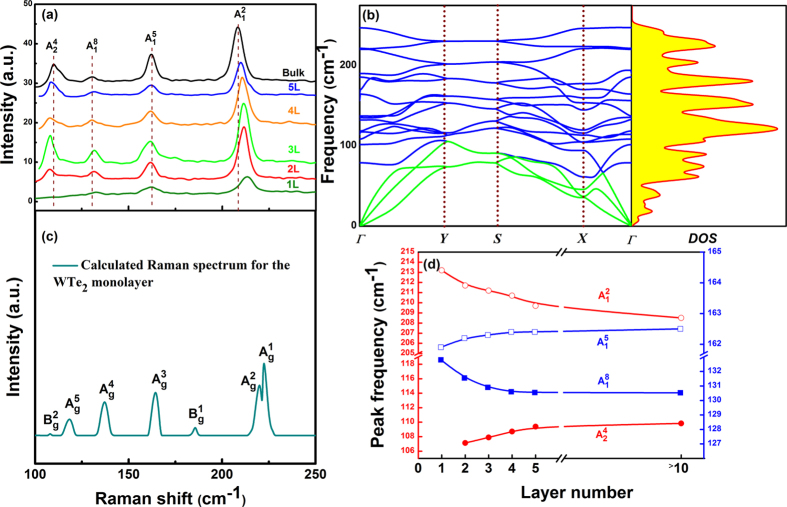
Raman evolution. (**a**) Raman spectra of bulk and few-layer WTe_2_. (**b**) Calculated phonon dispersion curve along the *Γ-Y-X-Γ* direction in the orthorhombic BZ, and DOS for the WTe_2_ monolayer. Blue and green curves are optical and acoustic vibrational branches, respectively. (**c**) Calculated Raman spectrum for the WTe_2_ monolayer. (**d**) Frequencies of the 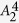, 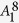, 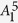 and 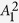 modes as functions of the layer number.

Figure 4a illustrates the Raman spectra of WTe_2_ as it evolved from bulk to monolayer. The four Raman active modes can be observed in the 2L, 3L, 4L and 5L samples. It is interesting to note that the 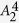 mode is absent in the 1L sample, which may make it convenient to use the Raman spectra to identify the WTe_2_ monolayer. The frequency difference between the 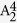 modes of the 2L sample and the bulk is about 2.7 cm^**−**1^. The 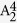 and 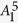 modes stiffen (blue-shift) in frequency as the number of layers increases, while the 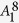 and 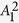 modes soften (red-shift). The 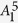 mode is the most stable and the displacement of its Raman shift, caused by the change in thickness, is less than 1 cm^**−**1^. In contrast, the 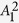 mode is found to be the most sensitive to the number of layers, and shifts up by about 4.8 cm^**−**1^ when the number of layers decreases to one.

With WTe_2_ evolving from bulk to monolayer, its point group changes from *C*_*2v*_ to *C*_*2h*_. The irreducible representation of the optical phonons in a WTe_2_ monolayer at the Brillion zone center (*Γ* point) is expressed as:


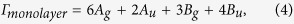


where 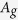 and 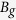 are Raman active; 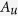 and 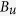 are infrared active. Only nine Raman-active modes are allowed in the WTe_2_ monolayer. This implies that some Raman peaks, observed in bulk, may be forbidden in the monolayer. Based on the group analysis, we calculated phonon dispersion curve in BZ, and DOS for WTe_2_ monolayer as shown in Fig. 4b. There are three acoustic (green curves) and fifteen optical (blue curves) phonon branches.

The vibrational modes of the WTe_2_ monolayer are exhibited in Table 2. To compare the vibrational modes of the bulk with those of the monolayer, we used the symmetry symbols of the *C*_*2v*_ and *C*_*2h*_ groups to name the corresponding modes, respectively. The atomic displacements of the Raman active modes were used to build a bridge between Raman modes of monolayer and bulk WTe_2_ as shown in Fig. 1b. Two of the vibrational modes in the bulk may correspond to the same modes in the monolayer. For example, the 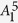 and 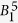 modes correspond to the 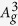 mode in Table 2, indicating that the 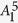 and 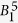 modes have the same atomic displacement as that of the 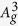 mode. For the 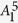 mode, the upper and lower atom layers exhibit the same phase of the atomic oscillations. But for the 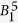 mode, the upper atom layer has an anti-phase with respect to the lower atom layer. Both of the modes can be distinguished in the multilayer samples (layer number ≥ 2), but turn into the 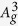 mode in the monolayer.

Our calculations show the 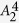 mode is forbidden in the monolayer, which consists with the experiments. Figure 4c shows the calculated Raman spectrum of the WTe_2_ monolayer. Only the 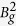 mode is a candidate to correspond to the 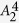 mode. However, Fig. 1b demonstrates that the atomic displacement of the 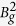 mode is different from that of the 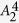 mode, but the same as that of the 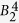 mode. Therefore, the 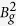 mode corresponds to the 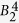 mode instead of the 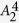 mode. On the basis of the theoretical analysis, the transition of point group from *C*_*2v*_ (bulk) to *C*_*2h*_ (monolayer) may be responsible for the absence of the 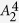 mode. In addition, the atomic displacement of the 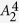 mode shows that only the half of tellurium atoms participate in oscillation, while the other half remains still (see Fig. 1b). In contrast to WTe_2_ bulk, all of the tellurium atoms participated in oscillation in the monolayer. This phenomenon may be attributed to the interlayer Van der Waals forces which are a dominant interaction between the adjacent atom layers. The loss of Van der Waals forces in the monolayer caused its atomic displacements to differ from those of bulk.

The frequencies of the four modes as a function of the number of layers are shown in Fig. 4d. The 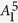 and 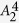 modes stiffen and the 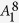, and 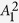 modes soften as the WTe_2_ monolayer evolves into a bulk. This implies that not only the interlayer Van der Waals coupling, but also stacking-induced structural changes or long-ranged Coulombic interlayer interaction may affect the atomic vibration^9^,^22^. If only Van der Waals coupling influenced the atomic vibrations, the 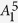, 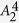, 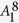 and 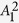 modes should all stiffen with layer number increasing^10^,^23^. The softening of the 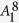, and 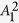 modes indicates that stacking-induced structural changes or long-ranged Coulombic interlayer interaction also plays an important role in the atomic vibration^9^,^10^,^22^. Our calculated results for the WTe_2_ monolayer confirm the shift directions of these vibrational modes.

In summary, we studied bulk and few-layer WTe_2_ using Raman spectroscopy. The six first-order Raman-active modes observed at room temperature differed from those of all other TMDs. DFPT and group theory were used to analyze the frequencies of the Raman peaks and their corresponding vibration modes. The Raman peaks were found to correlate with the optical phonons of 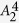, 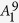, 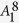, 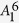, 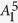 and 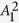. The 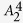 mode was forbidden in the monolayer due to the transition of the point group from *C*_*2v*_ (bulk) to *C*_*2h*_ (monolayer). As the number of layers increased, the 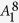, and 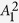 modes softened and the 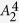 and 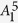 modes stiffened. Our study characterized all of the Raman peaks observed in bulk and few-layer WTe_2_ and provides a route to study the physical properties of 2D WTe_2_.

## Methods

The WTe_2_ single crystals were prepared using a chemical vapor transport method described by ref. 1. WTe_2_ flakes were mechanically exfoliated from a piece of bulk single crystal onto Si wafers covered with a 300-nm-thick SiO_2_ layer. The few-layer samples were first identified by optical microscopy, and then measured by atomic-force microscopy (AFM) to determine the thickness. Micro-Raman spectroscopy was used to analyze the WTe_2_ under ambient conditions. Non-polarized and off-resonance Raman measurements were performed with a 514.5-nm excitation laser. When the WTe_2_ flakes were investigated, the power of the excitation laser was reduced to 0.5 mW to obtain reliable Raman spectra against the oxidation. Our calculation and analysis were performed on basis of DFPT and group theory, using the experimental lattice parameters a = 3.496 Å, b = 6.282 Å and c = 14.07 Å and the same wavelength of the incident light as that in experiments^14^. The exchange correlation potential was represented by the local density approximation (LDA). The Brillouin zone integrations were performed with a 16×8×4 Monkhorst-Pack k-point mesh by using a plane-wave energy cutoff of 500 eV. All DFPT calculations model was appropriate for explaining the Raman spectrum using the CASTEP code^24^.

## Additional Information

**How to cite this article**: Jiang, Y. C. *et al*. Raman fingerprint for semi-metal WTe_2_ evolving from bulk to monolayer. *Sci. Rep.* **6**, 19624; doi: 10.1038/srep19624 (2016).

## Figures and Tables

**Table 1:** All of the possible optical phonons investigated through calculations and experiments.

Symmetry	Raman Activity	Infrared Activity	Experimental Frequency (cm^−1^)	Calculated Frequency (cm^−1^)
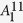	Y	Y		10.9
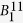	Y	Y		23.9
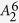	Y	N		29.7
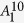	Y	Y		69.3
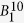	Y	Y		84.8
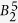	Y	Y		99.3
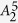	Y	N		103.9
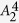	Y	N	109.8	110.0
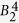	Y	Y		112.5
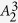	Y	N		114.0
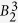	Y	Y		117.4
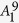	Y	Y	119.6	119.4
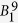	Y	Y		122.1
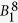	Y	Y		127.9
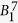	Y	Y		129.5
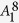	Y	Y	130.4	130.0
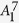	Y	Y		136.0
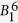	Y	Y		137.3
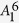	Y	Y	139.1	142.3
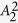	Y	N		160.0
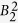	Y	Y		160.1
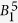	Y	Y		163.3
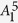	Y	Y	162.5	168.5
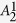	Y	N		180.1
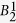	Y	Y		181.2
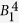	Y	Y		188.6
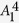	Y	Y		189.7
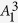	Y	Y		217.3
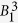	Y	Y		218.1
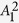	Y	Y	208.4	222.2
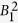	Y	Y		223.1
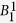	Y	Y		238.1
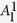	Y	Y		241.7

“Y” or “N” indicates that the chosen mode is allowed or forbidden.

**Table 2:** The calculated vibrational modes of the WTe_2_ monolayer.

Symmetry	Symmetry (bulk)	Experimental Frequency (cm^−1^)	Calculated Frequency (cm^−1^)
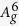	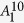 or 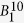		81.4
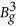	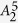 or 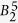		93.2
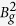	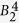		108.5
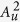	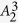 or 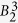		114.3
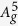	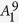		118.5
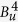	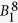		132.2
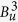	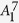 or 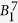		135.1
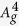	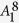 or 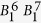	132.8	137.2
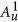	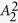 or 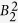		156.6
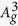	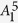 or 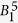	161.9	164.5
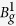	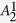 or 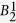		185.7
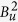	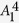 or 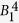		190.2
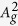	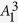		220.3
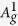	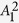	213.2	222.6
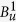	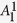 or 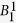		247.2
